# Perioperative Management of Estrogen Therapy As It Relates to Venous Thromboembolism in Aesthetic Surgery Patients

**DOI:** 10.1093/asjof/ojad027.004

**Published:** 2023-04-14

**Authors:** Bryn Morris, Renita Wilson, Victoria Aime, Max Shrout, Danielle Thornburg, Nicole DeLaPena, Shelley Noland, Edward Reece, Chad Teven

**Affiliations:** Mayo Clinic Arizona, Phoenix, AZ; Mayo Clinic Alix School of Medicine, Scottsdale, AZ; Metropolitan Plastic Surgery, Scottsdale, AZ; Mayo Clinic Arizona, Phoenix, AZ; Mayo Clinic Arizona, Phoenix, AZ; Mayo Clinic Alix School of Medicine, Scottsdale, AZ; Mayo Clinic Arizona, Phoenix, AZ; Mayo Clinic Arizona, Phoenix, AZ; Northwestern University, Chicago, IL

## Abstract

**Goals/Purpose:**

Perioperative management of estradiol therapy varies considerably among plastic surgeons performing aesthetic surgeries. This study aimed to review available literature regarding perioperative management of estradiol therapy in aesthetic surgery patients, and the risk it poses for developing VTE in the perioperative period.

**Methods/Technique:**

A literature search was performed using online databases to identify studies evaluating VTE in aesthetic surgery patients taking oral contraceptives (OCPs) or hormone replacement therapy (HRT), and transgender patients on feminizing hormone therapy undergoing non-flap feminization surgery (facial feminization and breast augmentation procedures). Reported outcomes were extracted from these studies and summarized.

**Results/Complications:**

A systematic review of the literature yielded 225 studies. Five of these studies met inclusion criteria. One study that was excluded, referenced an article that met inclusion criteria, which was ultimately included in our review for a total of six studies, representing 3,635 patients.

**Conclusion:**

Data regarding perioperative management of estrogen therapy is limited in the literature, and standardized guidelines for aesthetic surgery patients have not been elucidated. This has led to inconsistencies in how estrogen is managed for elective, cosmetic procedures. Further research is needed to determine standardized practice guidelines. In the meantime, case-by-case evaluation of surgical appropriateness should continue. Surgeons should also consider risk-mitigating strategies, such as a focus on patient education, more intentional patient questioning regarding estrogen-containing product use, a preoperative coagulability workup for patients with a personal or family history indicative of possible coagulopathy, and specialty-wide efforts to create an aesthetics database and evidence-based guidelines.

